# College Notes

**Published:** 1900-11

**Authors:** 


### COLLEGE NOTES.

At the Medical Department, University of Buffalo, the class of 1901 held its election in alumni hall, October 22, 1900, when the following-named officers were elected: R. E. P. Trevett, president; Roy G. Strong, vice-president; J. W. Reilly, secretary; W. W. Carleton, treasurer; T. M. Leonard, historian; J. A. Weidman, prophet; W. R. Paterson, orator; O. W. Steinlein, marshal; G. M. Hall, T. E. Spalding, G. H. Davis, J. B. Frisbee, executive committee.

---

The Dental Department of the University of Buffalo, in response to what appears to be an urgent need, proposes to organise a course of post-graduate bacteriological study, under the direction of Dr. William G. Bissell, to begin about the 1st of February, 1901, provided a sufficient number of practitioners will, in advance, enroll themselves as members of the class. It will not be attempted otherwise.

The instruction will be given in the evening, in the bacteriological laboratory of the college on Goodrich Street, and all the apparatus, microscopes, etc., belonging to it will be in use. The course will be about as follows: not less than six lectures and twelve evenings of laboratory work of two hours each, will be included. The lectures will be upon the following subjects:

(1) The bacteria—their general characteristics, morphology, chemical composition and vital phenomena; (2) Bacteriological technique, including the manufacture of media, the study of cultures, etc., etc.; (3) Infection and immunity; (4) Disinfection and sterilisation; (5) Bacterial pathology, general and special; (6) The hygiene of transmissible diseases.

The laboratory course will include the personal study and observation through methods of culture, staining, etc., of the most important organisms, including oral forms, pus-producing bacteria, bacillus of tetanus, bacillus tuberculosis, bacillus of diphtheria, bacillus typhosus, micrococcus of sputum, septicemia, etc., etc. A complete knowledge of the methods of proliferation with exhaustive studies of these and other organisms will be given.

Any one who desires to join such a class or to sustain such a course should, as soon as possible, communicate, either personally or by letter with the Dean, Dr. W. C. Barrett, at the college, as considerable time is necessary for the preparation of the material, and nothing will be done unless a sufficient number of practitioners offer themselves as members. Dr. Barrett's college hours are from 3 to 5 p.m.

This, we believe, is the first time an organised post-graduate course in bacteriology has been attempted, and we have no doubt of its success under the management of such an accomplished lecturer as Professor Bissell. The fee for the course is only $10, which is very reasonable, and brings it within the reach of all, leaving no excuse for declining the course on account of its expense.

